# Supplementary figures and images for: Telenomus nizwaensis (Hymenoptera: Scelionidae), an important egg parasitoid of the pomegranate butterfly Deudorix livia Klug (Lepidoptera: Lycaenidae) in Oman
Source: PLoS One. 2021 May 5;16(5):e0250464. doi: 10.1371/journal.pone.0250464 (PMC8099134; doi:10.1371/journal.pone.0250464)

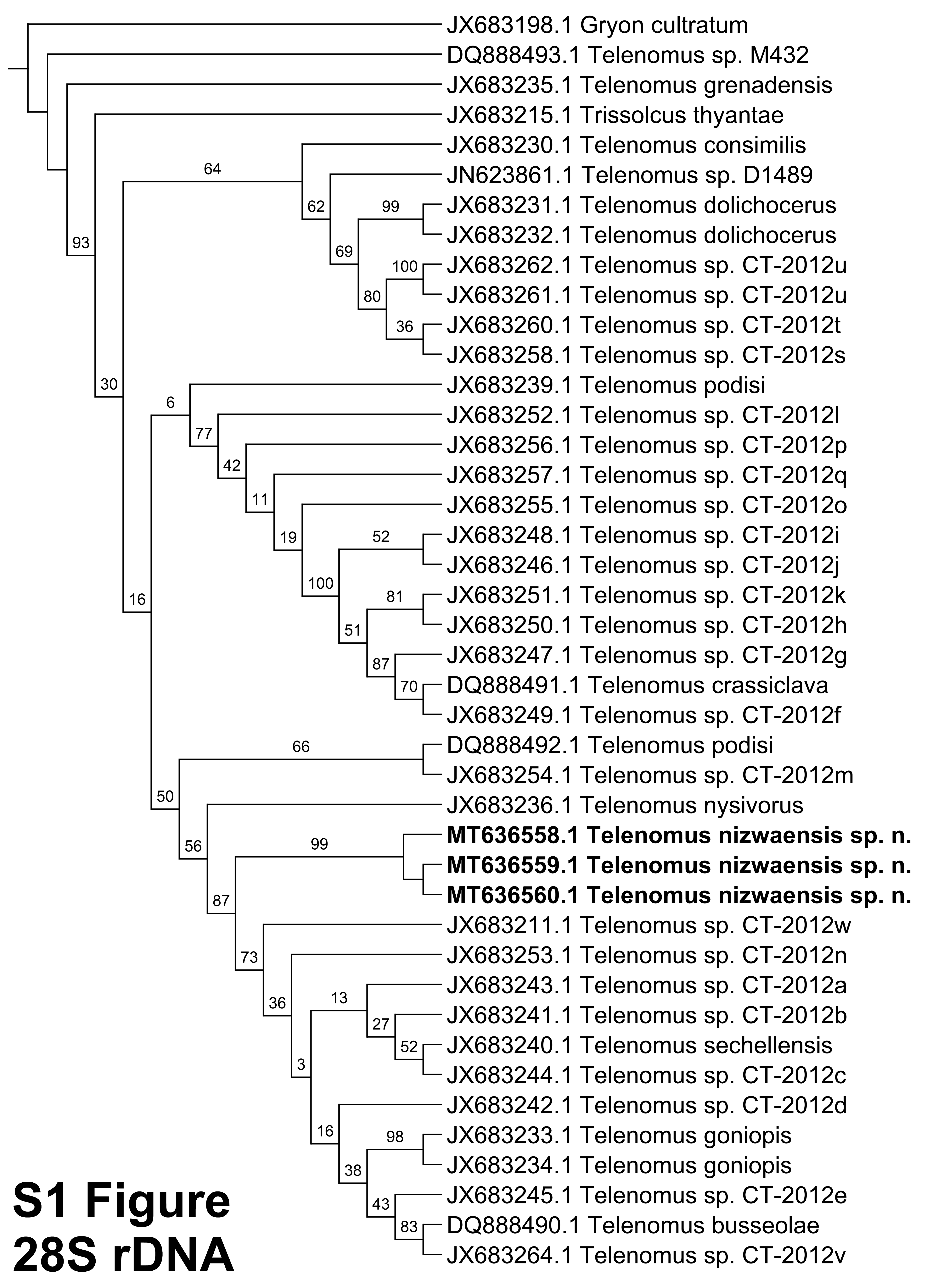

Supplement: S1 Fig — (TIF) [file pone.0250464.s001.tif]

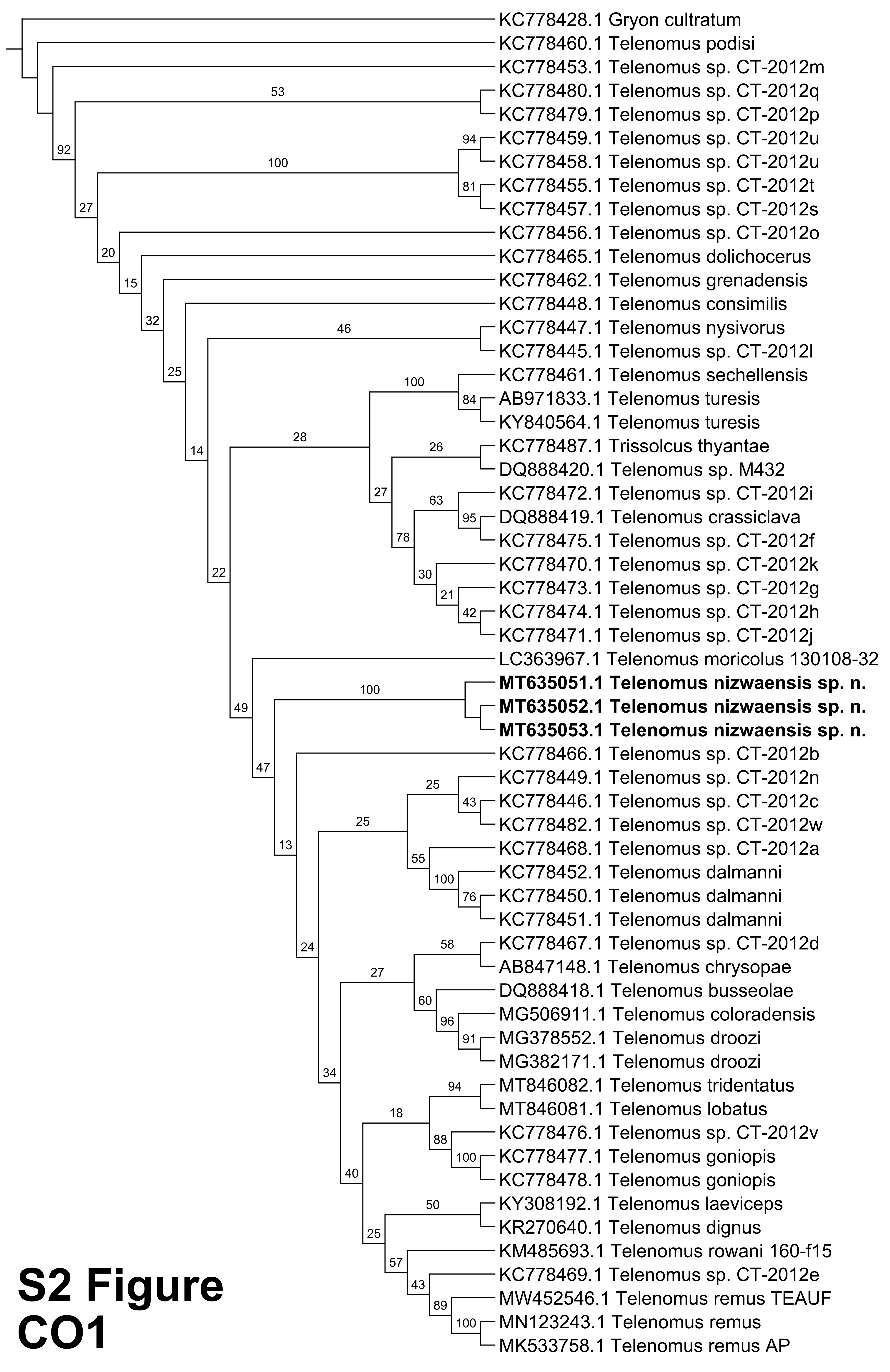

Supplement: S2 Fig — (TIF) [file pone.0250464.s002.tif]
