# Supplementary material for: Telenomus nizwaensis (Hymenoptera: Scelionidae), an important egg parasitoid of the pomegranate butterfly Deudorix livia Klug (Lepidoptera: Lycaenidae) in Oman
Source: PLoS One. 2021 May 5;16(5):e0250464. doi: 10.1371/journal.pone.0250464 (PMC8099134; doi:10.1371/journal.pone.0250464)
Supplement: Attachment — Submitted filename: Response to reviewers.docx [file pone.0250464.s006.docx]

PONE-D-20-34715
Telenomus nizwaensis (Hymenoptera: Scelionidae), an important egg parasitoid of the pomegranate butterfly Deudorix livia Klug (Lepidoptera: Lycaenidae) in Oman
PLOS ONE

RESPONSE TO REVIEWERS (our responses in blue text)

1. Please ensure that your manuscript meets PLOS ONE's style requirements, including those for file naming. The PLOS ONE style templates can be found at

<https://journals.plos.org/plosone/s/file?id=wjVg/PLOSOne_formatting_sample_main_body.pdf> and

<https://journals.plos.org/plosone/s/file?id=ba62/PLOSOne_formatting_sample_title_authors_affiliations.pdf>

2. We noticed you have some minor occurrence of overlapping text with the following previous publication, which needs to be addressed:
<https://journals.plos.org/plosone/article?id=10.1371%2Fjournal.pone.0223761>
In your revision ensure you cite all your sources (including your own work), and quote or rephrase any duplicated text outside the methods section. Further consideration is dependent on these concerns being addressed.

- Since the extraction + sequencing protocols, several imaging protocols and other aspects of the methods were exactly identical to those in previous publications, we see no reason to artificially modify them for the sake of making them read differently. However, we acknowledge that the previous +/- identical protocols should be cited. At the risk of over self-citation I have cited only <https://doi.org/10.1371/journal.pone.0223761> (Goniozus omanensis) and not <https://doi.org/10.1371/journal.pone.0230944> (Metaphycus macadamiae). Unfortunately this has necessitated re-numbering the references.

3. In your Methods section, please provide additional location information of the collection sites, including geographic coordinates for the data set if available.

- Done

4. Thank you for stating the following in the Acknowledgments Section of your manuscript:

"We thank the Ministry of Agriculture, Fisheries and Water Resources Agricultural Wealth,
304 Fisheries and Water Resources in Oman for support, especially Naser Al Abri and Issa Al-Mandhari
305 for their help in field work while collection and for offering other essential logistics. A. A-R was
306 funded by the Sultanate of Oman, Ministry of Higher Education, Research and Innovation under
307 grant number PGE055580 D.P.S/321/2017. We thank the Anglo-Omani Society for assistance
8
with publication charges. We also thank the Department of 308 Agricultural Development in Al Jabal
309 Al Akhdar for their valuable help, in particular as the contact point between us and local farmers."

We note that you have provided funding information that is not currently declared in your Funding Statement. However, funding information should not appear in the Acknowledgments section or other areas of your manuscript. We will only publish funding information present in the Funding Statement section of the online submission form.

Please remove any funding-related text from the manuscript and let us know how you would like to update your Funding Statement. Currently, your Funding Statement reads as follows:

"A. A-R: PGE055580 D.P.S/321/2017
Sultanate of Oman, Ministry of Higher Education, Research and Innovation
<https://www.mohe.gov.om>
The funders had no role in study design, data collection and analysis, decision to publish, or preparation of the manuscript."

Please include your amended statements within your cover letter; we will change the online submission form on your behalf.

- I have removed the specific funding references for the Oman Ministry. Should I also do this for the publication costs?

5. In your Data Availability statement, you have not specified where the minimal data set underlying the results described in your manuscript can be found. PLOS defines a study's minimal data set as the underlying data used to reach the conclusions drawn in the manuscript and any additional data required to replicate the reported study findings in their entirety. All PLOS journals require that the minimal data set be made fully available. For more information about our data policy, please see <http://journals.plos.org/plosone/s/data-availability>.

Upon re-submitting your revised manuscript, please upload your study’s minimal underlying data set as either Supporting Information files or to a stable, public repository and include the relevant URLs, DOIs, or accession numbers within your revised cover letter. For a list of acceptable repositories, please see <http://journals.plos.org/plosone/s/data-availability#loc-recommended-repositories>. Any potentially identifying patient information must be fully anonymized.

Important: If there are ethical or legal restrictions to sharing your data publicly, please explain these restrictions in detail. Please see our guidelines for more information on what we consider unacceptable restrictions to publicly sharing data: <http://journals.plos.org/plosone/s/data-availability#loc-unacceptable-data-access-restrictions>. Note that it is not acceptable for the authors to be the sole named individuals responsible for ensuring data access.

We will update your Data Availability statement to reflect the information you provide in your cover letter.

- We have submitted the unaligned and aligned fasta files to Dryad. Those files can be accessed at the following link: [https://doi.org/10.5061/dryad.sxksn0329](https://eur03.safelinks.protection.outlook.com/?url=https%3A%2F%2Fdoi.org%2F10.5061%2Fdryad.sxksn0329&data=04%7C01%7CA.Polaszek%40nhm.ac.uk%7Cedb6e7e973994d8fcee708d8c137d0f7%7C73a29c014e78437fa0d4c8553e1960c1%7C1%7C0%7C637471793181136012%7CUnknown%7CTWFpbGZsb3d8eyJWIjoiMC4wLjAwMDAiLCJQIjoiV2luMzIiLCJBTiI6Ik1haWwiLCJXVCI6Mn0%3D%7C1000&sdata=Olw1LZ54sCARU7hD0EWVmnlnJht4m5ebe6mOyisxzG8%3D&reserved=0).

6. PLOS requires an ORCID iD for the corresponding author in Editorial Manager on papers submitted after December 6th, 2016. Please ensure that you have an ORCID iD and that it is validated in Editorial Manager. To do this, go to ‘Update my Information’ (in the upper left-hand corner of the main menu), and click on the Fetch/Validate link next to the ORCID field. This will take you to the ORCID site and allow you to create a new iD or authenticate a pre-existing iD in Editorial Manager. Please see the following video for instructions on linking an ORCID iD to your Editorial Manager account: <https://www.youtube.com/watch?v=_xcclfuvtxQ>

- This should fine – I have been lead/corres[poning author on 2 PLoSOne papers since 2016

7. Please include captions for your Supporting Information files at the end of your manuscript, and update any in-text citations to match accordingly. Please see our Supporting Information guidelines for more information: [http://journals.plos.org/plosone/s/supporting-information](about:blank).

- Done

[Note: HTML markup is below. Please do not edit.]

Reviewers' comments:

Reviewer's Responses to Questions

**Comments to the Author**

1. Is the manuscript technically sound, and do the data support the conclusions?

The manuscript must describe a technically sound piece of scientific research with data that supports the conclusions. Experiments must have been conducted rigorously, with appropriate controls, replication, and sample sizes. The conclusions must be drawn appropriately based on the data presented.

Reviewer #1: Yes

Reviewer #2: Yes

2. Has the statistical analysis been performed appropriately and rigorously?

Reviewer #1: N/A

Reviewer #2: N/A

3. Have the authors made all data underlying the findings in their manuscript fully available?

The [PLOS Data policy](https://eur03.safelinks.protection.outlook.com/?url=http%3A%2F%2Fwww.plosone.org%2Fstatic%2Fpolicies.action%23sharing&data=04%7C01%7Ca.polaszek%40nhm.ac.uk%7C65c83cc933f0410b6b7308d8b352b815%7C73a29c014e78437fa0d4c8553e1960c1%7C1%7C0%7C637456515318819000%7CUnknown%7CTWFpbGZsb3d8eyJWIjoiMC4wLjAwMDAiLCJQIjoiV2luMzIiLCJBTiI6Ik1haWwiLCJXVCI6Mn0%3D%7C1000&sdata=j1IItv3tE4yOIrZ%2BZjugeJ%2FrM544cbpcqFruAZLbwEk%3D&reserved=0) requires authors to make all data underlying the findings described in their manuscript fully available without restriction, with rare exception (please refer to the Data Availability Statement in the manuscript PDF file). The data should be provided as part of the manuscript or its supporting information, or deposited to a public repository. For example, in addition to summary statistics, the data points behind means, medians and variance measures should be available. If there are restrictions on publicly sharing data—e.g. participant privacy or use of data from a third party—those must be specified.

Reviewer #1: Yes

Reviewer #2: Yes

4. Is the manuscript presented in an intelligible fashion and written in standard English?

PLOS ONE does not copyedit accepted manuscripts, so the language in submitted articles must be clear, correct, and unambiguous. Any typographical or grammatical errors should be corrected at revision, so please note any specific errors here.

Reviewer #1: Yes

Reviewer #2: Yes

5. Review Comments to the Author

Please use the space provided to explain your answers to the questions above. You may also include additional comments for the author, including concerns about dual publication, research ethics, or publication ethics. (Please upload your review as an attachment if it exceeds 20,000 characters)

Reviewer #1: In this manuscript, the authors describe a new species, Telenomus nizwaensis Polaszek sp. n., based on morphology and DNA sequence data. This egg parasitoid is an important biological agent against the pomegranate butterfly Deudorix (=Virachola) livia, which is a major pest of pomegranate in Oman. They also summarize the biology of the parasitoid species, which might enhance to the further use of this parasitoid in biological control programs. This study is quite straightforward and the manuscript is well written. There are a few points of consideration listed below that I think might improve the manuscript.
Major comments
1. In the method section, the authors say that eight individuals (4 females, 4 males) were subjected to DNA extraction (line 106), while in the result section they mentioned that 28S and COI were generated only from three specimens (line 208). So only these three specimens were successfully sequenced? What are the genders of these three specimens? It would be good to know that if these three specimens cover both sexes. Apparently, the studied specimens were reared from the eggs of the pomegranate butterfly from the same locality and it is mostly likely, the emerged parasitoids belong to the same species, but you never know, other species of Telenomus might present, too. If the sequenced specimens are both from female and male, then the association of both sexes can be sure.

This information has now been updated

2. The new species is placed in the Telenolmus californicus-group, I believe it would be useful to readers if the authors could provide more information on the diagnosis of this species group. Besides, since the new species has a 4-merous clava in the female, which is different from other species of the group, the placement in the group should be discussed more in more details. In other words, why do the authors believe this new species belong to the Telenolmus californicus-group?

- This point is very well-made. One of us (ZL) has thoroughly updated the discussion on the species-group placement after discussion with Prof. N. Johnson., the author of the “californicus complex”

3. The author stated that the CO1 sequence of the new species is best match to Telenomus dingus KR270640 (line 225), what is the identical percentage between these two species? And why the CO1 sequence of Telenomus dingus KR270640 was not included in the phylogenetic analysis?

- The analyses have been completed re-done. We included all available and appropriate sequnnces from GenBank as well as the sited sequence from Telenomus dignus, though that had to be extracted from a mitogenomic study. The results are much more detailed and rigorous, although conclusions from the first analysis are still completely supported. We have added a list of all sequences analysed, their depositories etc as supplementary files.

4. Line 95-96, the authors state “Observations of emergence from eggs of other hosts require confirmation”, also line 277-288 “Telenomus wasps that may have been T. nizwaensis have emerged from butterfly eggs infesting Acacia sp. pods (A. A-R. pers. obs.)”. The confirmation of the host range of the new species is important to biological control programs, it would be better to include the DNA of specimens emerged from other hosts into the analysis. Of course, this is optional if there were no such specimens available.

- Yes, that would me a “nice to have”, but considering how many 100s of undescribed Telenomus species there must be out there, simply not practically possible right now. At the very least, our thorough analysis shows that *T. nizwaenesis* does not cluster closely with any other known species.

Minor comments:
1. In figs 12-14, the first two trees indicated as RAxML, while the last is not, is it also RAxML?
2. Please provide the full descriptions for the abbreviations in figs 5-6, either in the main text or the legend.
3. It seems that there are duplicates of figs 9-10.

- All these points have been addressed

Reviewer #2: Telenomus is a large and difficult genus to study as the authors mentioned. The authors described a new parasitoid species with host information and DNA barcodes. The description of this new hymenopteran species will benefit the management of its host pest as a potential biological control agent, as well as facilitate the species delimitations/identifications of the genus Telenomus. This new species is placed in the californicus-group, however, the interpretation of this species group was not provided. Further, the sister grouping relationships were recovered in the phylogenetic analyses with limited data. However, the possibly close relationships of this new species with another species in the californicus-group were not discussed. The discussions about its close relationship with other species and the interpretation of the species group where it is placed could help readers to understand the systematic position of this species in this species group or even in this genus.

- All points well-made and addressed thoroughly in our revision – see also the points able

Please see the details as in the attached file.

6. PLOS authors have the option to publish the peer review history of their article ([what does this mean?](https://eur03.safelinks.protection.outlook.com/?url=https%3A%2F%2Fjournals.plos.org%2Fplosone%2Fs%2Feditorial-and-peer-review-process%23loc-peer-review-history&data=04%7C01%7Ca.polaszek%40nhm.ac.uk%7C65c83cc933f0410b6b7308d8b352b815%7C73a29c014e78437fa0d4c8553e1960c1%7C1%7C0%7C637456515318819000%7CUnknown%7CTWFpbGZsb3d8eyJWIjoiMC4wLjAwMDAiLCJQIjoiV2luMzIiLCJBTiI6Ik1haWwiLCJXVCI6Mn0%3D%7C1000&sdata=QoSgb7vEFTWefZT%2BScJwR6qZ4Zzt59bd7PBExEp6VeM%3D&reserved=0)). If published, this will include your full peer review and any attached files.


If you choose “no”, your identity will remain anonymous but your review may still be made public.


**Do you want your identity to be public for this peer review?** For information about this choice, including consent withdrawal, please see our [Privacy Policy](https://eur03.safelinks.protection.outlook.com/?url=https%3A%2F%2Fwww.plos.org%2Fprivacy-policy&data=04%7C01%7Ca.polaszek%40nhm.ac.uk%7C65c83cc933f0410b6b7308d8b352b815%7C73a29c014e78437fa0d4c8553e1960c1%7C1%7C0%7C637456515318828991%7CUnknown%7CTWFpbGZsb3d8eyJWIjoiMC4wLjAwMDAiLCJQIjoiV2luMzIiLCJBTiI6Ik1haWwiLCJXVCI6Mn0%3D%7C1000&sdata=Z3jHQinZKkFrXfjMSZw4EEoQnfAQT1AJlpHgQqOSRNs%3D&reserved=0).

Reviewer #1: No

Reviewer #2: No

[NOTE: If reviewer comments were submitted as an attachment file, they will be attached to this email and accessible via the submission site. Please log into your account, locate the manuscript record, and check for the action link "View Attachments". If this link does not appear, there are no attachment files.]

While revising your submission, please upload your figure files to the Preflight Analysis and Conversion Engine (PACE) digital diagnostic tool, [https://pacev2.apexcovantage.com/](https://eur03.safelinks.protection.outlook.com/?url=https%3A%2F%2Fpacev2.apexcovantage.com%2F&data=04%7C01%7Ca.polaszek%40nhm.ac.uk%7C65c83cc933f0410b6b7308d8b352b815%7C73a29c014e78437fa0d4c8553e1960c1%7C1%7C0%7C637456515318828991%7CUnknown%7CTWFpbGZsb3d8eyJWIjoiMC4wLjAwMDAiLCJQIjoiV2luMzIiLCJBTiI6Ik1haWwiLCJXVCI6Mn0%3D%7C1000&sdata=khFvUI9z%2BODf83aCBW7VsGwROV8R%2BTrv9fni47svZa4%3D&reserved=0). PACE helps ensure that figures meet PLOS requirements. To use PACE, you must first register as a user. Registration is free. Then, login and navigate to the UPLOAD tab, where you will find detailed instructions on how to use the tool. If you encounter any issues or have any questions when using PACE, please email PLOS at [figures@plos.org](mailto:figures@plos.org). Please note that Supporting Information files do not need this step.
